# Correction of Alar Base Retraction by Levator Labii Alaeque Nasi Muscle Dissection and Alar Rim Grafting: A Clinical Prospective Study

**DOI:** 10.7759/cureus.34184

**Published:** 2023-01-25

**Authors:** Yazeed Alghonaim, Tarfah AlSayyari

**Affiliations:** 1 Otolaryngology, King Saud Bin Abdulaziz University for Health Sciences, Riyadh, SAU; 2 College of Medicine/Surgery, King Saud Bin Abdulaziz University for Health Sciences, Riyadh, SAU

**Keywords:** alar base retraction, levator labii alaque nasi muscle, nasal base asymmetry, levator labii alaque nasi, llanm, cephalic elevation of the alar margin

## Abstract

Alar base retraction may result in disharmony of nose structures. Although correction of this retraction may play an important role in patient satisfaction, there is a limited number of studies on alar base retraction correction. The aim of this study was to manage alar base retraction with minimal undesirable results. Six patients underwent correction of alar base retraction using dissection of the levator labii alaque nasi muscle with or sometimes without alar rim grafting. Assessment of the defect was done using preoperative and postoperative frontal view photographs of each patient. Comparing the preoperative and postoperative photographs shows a significant improvement in the nasal base asymmetry, and all six patients have aesthetically satisfying results after a 12-month follow-up period. In conclusion, nasal base retraction is a well-known deformity that has been an area of focus in the rhinoplasty field and management of this defect has very promising results.

## Introduction

Muscles are essential elements in nose dynamic anatomy as well as facial expressions [1,2]. Four major muscle groups are responsible for positioning the nasal base: depressors, elevators, dilators and compressors. One of the important muscles in the nasal base is the levator labii alaque nasi muscle (LLANM). LLANM has two insertions: medially into the lateral nostril and laterally into the upper lip, in which the lateral part raises and evert the upper lip, while the medial part retracts the alar rim laterally and superiorly to dilate the nostrils. This muscle is innervated by the zygomatic and superior buccal branches of the facial nerve and supplied by the facial artery and the infraorbital branch of the maxillary artery [3,4].

Alar base retraction is defined as a cephalic elevation of the alar margin, which may result in excess nostril show and aesthetically unsatisfied surgical results [3]. In order to correct that, there are some excisional and repositioning procedures that can be done; however, these procedures may result in nostril distortion or stenosis as well as elevating the upper lip instead of releasing the alar base caudally if corrected with excisional procedures [5]. Therefore, up until now there is no universally accepted surgical technique for alar base retraction correction [5].

## Technical report

In the period from June 2018 to June 2019, six patients underwent rhinoplasty with correction of nasal base retraction by dissection of the levator labii alaque nasi muscle with or in some cases without alar rim grafting, with a 12-month postoperative follow-up. All surgeries were performed by the same surgeon (AlG. Y). The study was approved by the institutional review board at King Abdullah International Medical Research Center (KAIMRC), Riyadh, Saudi Arabia (approval RC20/068/R), and written informed consent was obtained from all the patients.

Diagnoses were made by preoperative analysis of the frontal and basal view pictures (Figures 1, 2). Measurement in millimeters of alar base retraction was taken preoperatively and one month postoperatively. Preoperative and postoperative photographs of each patient were taken from a frontal view, as well as a frontal view of the patient smiling. Two independent certified plastic surgeons evaluated the preoperative and postoperative photographs. Exclusion criteria were patients with acquired alar base retraction.

**Figure 1:**
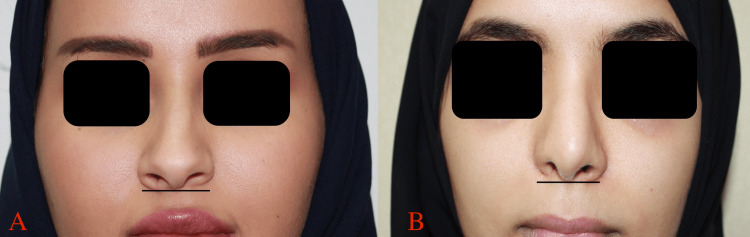
Preoperative analysis of alar base retraction with frontal view, A: patient A, B: patient B.

**Figure 2:**
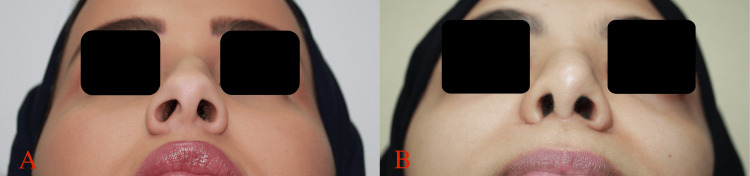
Preoperative analysis of alar base retraction with basal view. A: patient A, B: patient B.

For the operative technique, rhinoplasty was performed under general anesthesia. Alar base retraction was corrected at the end of the operation.

Dissection of the LLANM starts with the subcutaneous tissue and making an internal lateral incision. After that, dissection continues and a thin superficial musculoaponeurotic system (SMAS) layer is exposed and incised. Subsequently, lateral to the pyriform rim, a sharp dissection will show the LLANM. After cauterization, transection of the muscle is made with a scissor (Video 1). 

**Video 1:** Dissection of levator labii alaque nasi muscle.

Results

Six patients underwent rhinoplasty. Five of them had open technique while one patient underwent closed rhinoplasty. LLANM dissection was done in all the patients and two of them had alar rim graft placement. 

Postoperatively, patients were followed up for 12 months. Early postoperative period was uneventful for all patients, and none of them experienced any nasal distortion or recurrence of nasal deformity in the long-term follow-up period (Figures 3, 4, 5, 6).

**Figure 3:**
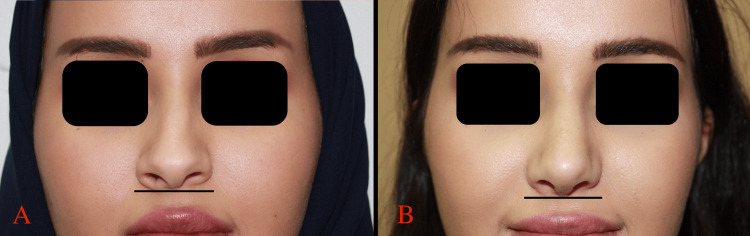
A: Patient A preoperative frontal view, B: Patient A one month postoperative frontal view.

**Figure 4:**
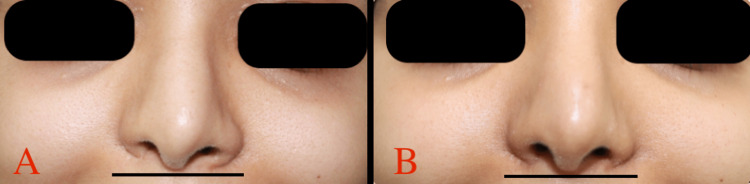
A: patient B preoperative, B: patient B one month postoperative.

**Figure 5:**
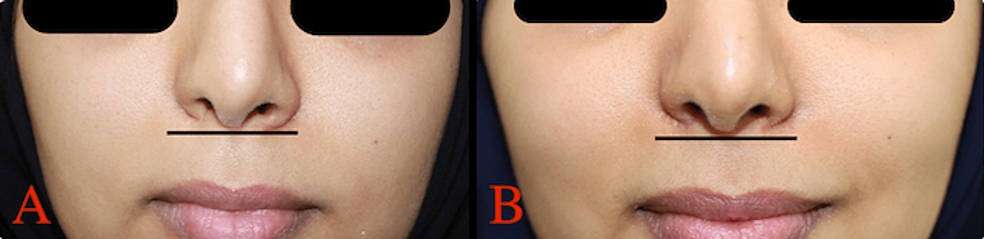
A: patient B preoperative frontal view, B: patient B one month postoperative frontal view.

**Figure 6:**
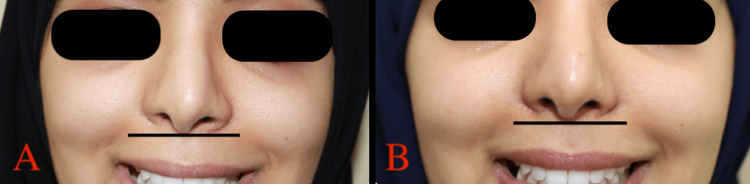
A: Preoperative frontal view of patient B smiling, B: one month postoperative frontal view of patient B smiling.

## Discussion

Alar base retraction is a retraction of the horizontal part of the alar sulcus cephalically. It is usually confused with alar retraction, which is characterized by more than 2 mm alar rim retraction in the long axis of the alar rim to the nostril [3,6].

The function of the levator labii alaque nasi muscle in elevating the alar base during facial expression was first introduced by Pessa and Pessa and Brown in cadaver studies [2,4]. After that, Pessa dissected the LLANM unilaterally to improve the medial nasolabial fold in older patients and to correct the nasal asymmetry as a result of facial paralysis via subciliary incision [4,7].

One of the techniques that have been tested on humans for correcting alar base retraction is the seesaw technique by Hyun et al. It was used to correct vertical alar discrepancy [8]. This technique is an excisional procedure, which has some disadvantages such as visible scar formation, diminished vascular supply, nostril distortion, or stenosis. Toriumi, on the other hand, used cartilage grafting in order to fix the projection problem in the premaxilla [9]. He also corrected the vertical alar deficiency with a lateral crural release and strut graft placement [9]. Because this method tends to widen the nasal base, it is appropriate for patients with a narrow nasal base [10]. Another study by Gruber et al., with a total number of 12 patients, reported a reduction in the retraction with a combination of alar base release and different excisional procedures based on the cause of the increased width [11].

A recent study by Tas et al. used a treatment algorithm for alar base retraction in which all of the patients had LLANM dissection and only patients with a deviated or crooked nose or alar base retraction more than 2 mm had rim grafting [12] while in this study, grafting was done only for people with external valve weakness. 

In this report, LLANM transection has shown to be a successful treatment of alar base retraction in long-term follow-up, and no recurrences of dorsal deviation or alar base retraction were observed. These successful results encouraged us to seek more studies with the same approach for alar base retraction.

Although the results are promising, it has its limitation since it is based on a single surgeon’s experience. There is decidedly a need for further studies with larger sample sizes to fully understand the impact of an LLANM transection and alar rim grafting in alar base retraction.

## Conclusions

Nasal base retraction is a well-known deformity and has been a hot research topic in the rhinoplasty field. Management of such defects requires a careful analysis and proper selection of the surgical technique to get aesthetically desired results.

## References

[1] Rohrich RJ, Hoxworth RE, Thornton JF, Pessa JE (2008). The pyriform ligament. Plast Reconstr Surg.

[2] Pessa JE, Brown F (1992). Independent effect of various facial mimetic muscles on the nasolabial fold. Aesthetic Plast Surg.

[3] Taş S (2016). Correcting the alar base retraction in crooked nose by dissection of levator alaque nasi muscle. Ann Plast Surg.

[4] Pessa JE (1992). Improving the acute nasolabial angle and medial nasolabial fold by levator alae muscle resection. Ann Plast Surg.

[5] Ponsky D, Guyuron B (2010). Alar base disharmonies. Clin Plast Surg.

[6] Gunter JP, Rohrich RJ, Friedman RM (1996). Classification and correction of alar-columellar discrepancies in rhinoplasty. Plast Reconstr Surg.

[7] Pessa JE, Crimmins CA (1993). The role of facial muscle resection in reconstruction of the paralyzed face. Ann Plast Surg.

[8] Hyun SM, Medikeri GS, Jung DH (2015). The seesaw technique for correction of vertical alar discrepancy. Plast Reconstr Surg.

[9] Cheon YW, Park BY (2010). Long-term evaluation of elongating columella using conchal composite graft in bilateral secondary cleft lip and nose deformity. Plast Reconstr Surg.

[10] Toriumi DM (2015). Discussion: the seesaw technique for correction of vertical alar discrepancy. Plast Reconstr Surg.

[11] Gruber RP, Freeman MB, Hsu C, Elyassnia D, Reddy V (2009). Nasal base reduction: a treatment algorithm including alar release with medialization. Plast Reconstr Surg.

[12] Tas S, Colakoglu S, Lee BT (2017). Nasal base retraction: a treatment algorithm. Aesthet Surg J.

